# Aggressive phenotype editing by modulated immune cells

**DOI:** 10.1192/j.eurpsy.2022.278

**Published:** 2022-09-01

**Authors:** E. Markova, E. Serenko, M. Knyazheva

**Affiliations:** Federal State Research Institute of Fundamental and Clinical Immunology, Neuroimmunology Lab., Novosibirsk, Russian Federation

**Keywords:** aggression, immune cells

## Abstract

**Introduction:**

In human society increased aggressiveness is one of the main social and health problems. Immune cells have a regulatory effect on the central nervous system functions, including regulation of behavior.

**Objectives:**

The aim of the study was to investigate the effect of *in vitro* neuroleptic-modulated immune cells transplantation on behavioral phenotype and brain cytokines in aggressive syngeneic recipients.

**Methods:**

(CBAxC57Bl/6) F1 aggressive male mice, developed in conditions of social confrontation, were undergoing the transplantation of syngeneic immune cells with *in vitro* chlorpromazine-modulated functional activity. Recipient’s behavioral phenotyping was performed using modern hardware and software complex EthoVision XT. The brain cytokines content was assessed by ELISA.

**Results:**

It was found that repeated experience of aggression, accompanied by victories, leads to a change in male mice behavior, which manifests itself by increased motor activity, irritability, severe anxiety, and the appearance of stereotypies. Transplantation of chlorpromazine-modulated splenocytes in aggressive recipient was accompanied by decreased motor activity in the Open Field, increased open arm activity in Plus Maze, reflects anti-anxiety behavior; decreased time spent close to the partition and the total duration of attacks after removal of the partitionin in resident-intruder test, reflects decreased aggressive motivation. Behavioral changes in recipients were accompanied with cytokines brain changes: decreased IL-1β, IL-2, IL-6, INFγ in the hippocampus; increased IL-4 and decreased INFγ in the hypothalamus; decreased IL-1β in the frontal cortex.

**Conclusions:**

Chlorpromazine - modulated immune cells have a positive aggressive behavior editing effect being involved in the central mechanisms underlying the development of aggressive reactions.

**Disclosure:**

No significant relationships.

